# A workplace intervention program and the increase in HIV knowledge, perceived accessibility and use of condoms among young factory workers in Thailand

**DOI:** 10.1080/17290376.2017.1387599

**Published:** 2017-10-16

**Authors:** Aphichat Chamratrithirong, Kathleen Ford, Sureeporn Punpuing, Pramote Prasartkul

**Affiliations:** ^a^ Institute for Population and Social Research, Mahidol University, Salaya, Nakhonpathom 73170, Thailand; ^b^ School of Public Health, University of Michigan, Ann Arbor, MI 48104, USA

**Keywords:** factory workers, workplace intervention, regular sexual partner, condom vending machine, HIV/AIDS prevention, Thailand, travailleur d’usine, intervention sur le lieu de travail, partenaire sexuel régulier, distributeur de préservatifs, VIH/SIDA prévention, Thaïlande

## Abstract

Vulnerability to Human Immunodeficiency Virus (HIV) infection among factory workers is a global problem. This study investigated the effectiveness of an intervention to increase AIDS knowledge, perceived accessibility to condoms and condom use among young factory workers in Thailand. The intervention was a workplace program designed to engage the private sector in HIV prevention. A cross-sectional survey conducted in 2008 to measure program outcomes in factories in Thailand was used in this study. The workplace intervention included the development of policies for management of HIV-positive employees, training sessions for managers and workers, and distribution of educational materials and condoms. A multi-level analysis was used to investigate the effect of HIV/AIDS prevention program components at the workplace on HIV/AIDS knowledge, perceived accessibility to condoms and condom use with regular sexual partners among 699 young factory workers (aged 18–24 years), controlling for their individual socio-demographic characteristics. Interventions related to the management and services component including workplace AIDS policy formulation, condom services programs and behavioral change campaigns were found to be significantly related to increased AIDS knowledge, perceived accessibility to condoms and condom use with regular partners. The effect of the HIV/AIDS training for managers, peer leaders and workers was positive but not statistically significant. With some revision of program components, scaling up of workplace interventions and the engagement of the private sector in HIV prevention should be seriously considered.

## Résumé

La vulnérabilité au virus de l'immunodéficience humaine (VIH) chez les travailleurs d'usine est un problème mondial. Cette étude a examiné l'efficacité d'une intervention visant à accroître les connaissances sur le sida, l'accessibilité perçue aux préservatifs et l'utilisation du préservatif chez les jeunes travailleurs d'usine en Thaïlande. L'intervention était un programme sur le lieu de travail conçu pour engager le secteur privé dans la prévention du VIH. Une enquête transversale menée en 2008 pour mesurer les résultats du programme dans les usines en Thaïlande a été utilisée dans cette étude. L'intervention sur le lieu de travail comprenait l'élaboration de politiques pour la gestion des employés séropositifs, des séances de formation pour les gestionnaires et les travailleurs et la distribution de matériel éducatif et de préservatifs. Une analyse à plusieurs niveaux a été utilisée pour étudier l'effet des composantes du programme de prévention du VIH/SIDA sur le lieu de travail sur les connaissances sur le VIH/SIDA, l'accessibilité perçue aux préservatifs et l'utilisation du préservatif avec des partenaires sexuels réguliers parmi 699 jeunes travailleurs d'usine (18–24 ans), en tenant compte de leurs caractéristiques sociodémographiques individuelles. Les interventions liées à la composante gestion et services, y compris la formulation des politiques de lutte contre le SIDA en milieu de travail, les programmes de services de préservatifs et les campagnes de changement comportemental, sont significativement liées à l'augmentation des connaissances sur le SIDA. L'effet de la formation sur le VIH/SIDA pour les gestionnaires, les représentants du personnel et les travailleurs était positif mais pas statistiquement significatif. Avec une certaine révision des composantes du programme, l'intensification des interventions sur le lieu de travail et l'engagement du secteur privé, une meilleure prévention du VIH pourra être sérieusement envisagée.

## Introduction

Vulnerability to Human Immunodeficiency Virus (HIV) infection among factory workers is a global problem. Workers are often young adults who are away from their families. Studies conducted in several countries have documented high risk behaviors in this population (Borgdorff et al., 1994; Buregyeya, Bazeyo, Moen, Michelo, & Fylkesnes, 2008; Cash, Sanguansermsri, Busayawong, & Chuamanochan, 1997; Kumar et al., 2009; Mukoka et al., 1991; Nishigaya, 2002; Puri & Cleland, 2006). For examples, Puri and Cleland (2006) found very low use of condoms despite a high level of AIDS knowledge among the factory workers of Nepal. Nishigaya (2002) found that female factory workers in Cambodia rarely used condoms with their regular partners. In a study of migrant and non-migrant factory workers in Kolkata, condom use was very low, particularly among the migrants (Kumar et al., 2009). Another study conducted among urban factory workers in Tanzania has found that condom use with casual partners was highest, but with a regular partner (spouse) was very low (Borgdorff et al., 1994). The vulnerability of factory workers to HIV infection is related to their young age as well as their distance from their families (Cash et al., 1997).

The epidemiology of HIV infection in Thailand has changed since the implementation in the 1990s of the 100% condom use program (Rojanapithayakorn & Hanenberg, 1996) that aimed to increase condom use in commercial sex transactions. The major factor related to increasing HIV infection at that time was through commercial sex. Currently, epidemiological surveillance indicates that new HIV and STI infections continue to increase among Thai youth (National AIDS Prevention and Alleviation Committee, Ministry of Public Health of Thailand, 2010). However, commercial sex has become a less common source of infection because of the successful high rate of condom use advocated by the 100% condom use program, while infection through non-commercial partners and regular partners among whom condom use is very low, has become more crucial. Recent HIV surveillance data show an increase in HIV prevalence among pregnant women and military conscripts (National AIDS Prevention and Alleviation Committee, Ministry of Public Health of Thailand, 2010; Bureau of Epidemiology, 2004). The estimate of new HIV infections in Thailand is more than 10,000 cases every year (Bureau of Epidemiology, 2004). The epidemic in Thailand has become generalized rather than concentrated. In spite of that, the prevention effort by the public sector declined after the country’s financial crisis in the late 1990’s. By 2003, only 8% of the HIV/AIDS budget was for prevention (United Nations Development Program – UNDP, 2004).

The Thai Ministry of Labor, working with the Thai Business Coalition on AIDS (TBCA) promoted a scaling up of AIDS programs in the workplace to increase the role of the private sector in prevention and management of AIDS activities. This program developed the organization standard for managing HIV in the workplace, namely the AIDS-response Standard Organization (ASO) (National AIDS Prevention and Alleviation Committee, Ministry of Public Health of Thailand, 2010). The strength of the private sector model for workplace programs for HIV/AIDS prevention has been clearly recognized (Baker, Allen-Toland, & Graham, 2006; UNDP, 2004). With financial assistance from the Global Fund (GFATM) and administrative support from the Ministry of Labor, the TBCA implemented this workplace intervention. As of 2008, a total of over 11,000 worksites with nearly 3 million employees were reached (National AIDS Prevention and Alleviation Committee, Ministry of Public Health of Thailand, 2010).

The results of the program showed an increase in AIDS policy-related activities in the workplace and a rise in positive attitudes and perceptions about HIV/AIDS among employees (National AIDS Prevention and Alleviation Committee, Ministry of Public Health of Thailand, 2010). However, the evidence for change in condom use among employees was not adequately documented. Moreover, the level of condom use was very low among regular partners compared to non-regular and commercial partners (Chamratrithirong, Kittisuksathit, Podhisita, Isarabhakdi, & Sabaiying, 2006), In accordance with the generalized epidemic in Thailand, consideration of promotion in condom use among factory workers with regular sexual partners is needed.

The objective of this study was to investigate the effect of the workplace program intervention on AIDS knowledge, perceived accessibility to condoms and condom use with regular partners among young factory workers in Thailand. The conceptual framework for the study was based on constructs from the Health Belief Model (Glanz, Rimer, & Lewis, 2002; Janz & Becker, 1984). This theory assumes that behavior is influenced by perceived susceptibility to the disease as well as positive and negative beliefs about health behaviors. Programmatic activity can provide cues to action for these health behaviors. Hence, we hypothesize that the programmatic factors will have positive effects on preventive behaviors. The aims are to determine which components of the intervention program affected AIDS knowledge, perceived accessibility to condoms and condom use.

## Data

Data were drawn from a cross-sectional behavioral survey of factory workers conducted in 2008. Twenty-eight factories participating voluntarily in the TBCA project were selected and included in the study. Eighteen factories were first selected with the probability of selection proportional to factory size (PPS method) from the list of 240 TBCA targeted factories of 300 or more workers. However since the number of young workers in certain factories was not enough to have a fair sample size for the study, 10 more factories were selected from the TBCA list of factories to insure an adequate sample of young workers. The recruitment of respondents was done with the assistance of factory’s management as it fits into the work schedule of the workers. The selection was based only on the work schedule and not on workers’ behavior or characteristics. In total, 699 young workers aged 18–24 in 28 factories were interviewed. Among these, 520 respondents were having sex with regular partners and were included in the analysis of condom use. This study received approval from IRB Committee of the Institute for Population and Social Research, Mahidol University.

## Intervention description

A pilot project was launched in Thailand to develop a policy tool for effective prevention and management of HIV/AIDS in the private sector workplace setting. The theoretical basis for the program was the Health Belief Model (Glanz et al., 2002; Janz & Becker, 1984). Accordingly, the program aimed to increase perceived susceptibility to HIV infection, to promote positive health beliefs about HIV prevention and to remove barriers towards preventive behaviors such as condom use. Based in the workplace, the program also addressed issues related to the welfare of HIV-positive workers in the factories. The project was implemented via a tri-partite arrangement between government, non-government and private sector agencies. The TBCA contacted a network of non-governmental organizations (NGOs) to implement the HIV program. The program consisted of several components as follows:

1. *ASO certification*. The program assisted factories in obtaining ASO certification from the government. For certification, a factory must have a non-discriminatory policy on HIV-positive employees, a documented HIV/AIDS policy, confidentiality procedures with regard to HIV-positive employees, support and care programs for staff affected with HIV, HIV/AIDS education in the workplace, and participation in community projects for the prevention and control of HIV/AIDS and related problems.
2. *Training.* Training programs were held at least once per year. First, manager training was conducted at a provincial office. This three-hour training consisted of basic facts and work place policy on HIV, CD4 counts, opportunistic infections, and Antiretroviral (ARV) drug in order to understand how HIV is treated and how to live and work with HIV-positive persons, an HIV transmission risk game, awareness of discrimination and issues concerning voluntary blood testing. The program also covered the National Code of Practice on HIV/AIDS in the Workplace, ASO indicators and criteria. Also, one or two persons, being selected as peer leaders, were sent from each factory to a regional two-day training program consisting of in-depth knowledge of HIV, CD4 counts, ARV, post-exposure prophylaxis (PEP), mother to child transmission, HIV testing, care and support, attitudes adjustment, and a risk-group exercise. Other topics included how to deal with sensitive issues, how to give advice, how to answer HIV-prevention questions, issues to consider as an HIV/AIDS trainer, and the HIV/AIDS policy of the organization. Third, a 3-hour, in-factory training program for 50 workers was included and covered basic facts about HIV, CD4 counts, opportunistic infections and ARV to understand how HIV is treated and how to live and work with HIV-infected persons. The training also included an HIV transmission game, a risk level game, condom use, awareness of discrimination, voluntary blood testing and the importance of an HIV/AIDS work place policy. The training was conducted for only 50 workers due to the limited factory budget and the TBCA program design to be cost effective with the expectation of diffusion among workers.
3. *Condom distribution* was implemented in some factories, including a free condom distribution program and the installation of condom vending machines to increase access to condoms among employees.
4. *Mobile HIV/AIDS exhibitions* were staged by the program at some factories upon request. Mini HIV/AIDS exhibitions were distributed to every company. A manager’s handbook, a trainer’s handbook and an employee’s handbook were distributed to every training participant.

## Variable measurement

### Program level measurement

Data on the four program activities mentioned above were collected by interviews conducted with factory managers at the factory sites. ASO certification was measured by a dummy variable (1 = had certification, 0 = did not have certification), the three levels of training (managers, peer leaders and workers) were each measured by a dummy variable (1 = had training, 0 = did not have training), each of the two condom service programs was measured by a dummy variable (1 = had free condom distribution program, 0 = did not have free condom distribution program; and 1 = had condom machine installed, 0 = did not have condom machine installed), and the campaign activity was also measure by a dummy variable whether the factory organized to have the mobile HIV/AIDS exhibition or not (1 = yes, 0 = no). In total, the program variables at the factory level included seven dummy variables.

### Individual level measurement

Individual characteristics of workers including age, gender, marital status and education were used as control variables in this study. Age was measured in single years from 18 to 24. Gender was measured as male (1) and female (0). Marital status was measured as married (1) or not married (0). Education was measured with several dichotomous variables including diploma level, and high school level or less. One of the dependent variables (AIDS knowledge) was measured by summing the number of correct responses to nine questions about AIDS prevention and transmission. These nine questions include knowledge on HIV transmission (by having meals together, mosquito bites, sharing needles, sexual intercourse, anal intercourse, mother to child transmission), HIV protection (use of condoms, having one faithful partner, and misconceptions about the risk of sex with a healthy-looking person). The alpha reliability coefficient for the scale was 0.76. Perceived condom accessibility was measured by asking respondents if they know a place where condoms are provided (1 = yes, 0 = no).

Respondents were asked whether their current sexual partners were regular partners, regardless of their marital status as single, married or living together. Condom use with these regular partners was the dependent variable. Condom use with regular partners was measured by condom use at the last episode of sex with their regular partner within the last year (1 = yes, 0 = no).

## Statistical methods

In order to investigate the effectiveness of the program intervention, the researchers focused on the degree or the intensity of the seven components of the intervention. The seven program input variables in the workplace included meeting the ASO criteria and receiving ASO Certification, implementing three levels of training activities (training of managers, peer leaders and workers), having two condom distribution activities (free condom service program and installation of condom machine in the workplace), and deploying the mobile HIV/AIDS exhibition. Table 1 presents the distribution of these seven HIV/AIDS prevention program interventions among the 28 factories under study. As high as about 60% of the 28 factories had received ASO certification. Other interventions were conducted by only half of the 28 factories or less. For example, HIV/AIDS training for managers was carried out in only about 30% of the factories.

**Table 1.** Distribution of the 7 HIV/AIDS prevention program interventions among the 28 factories under study in 2008.

Interventions	Number of factories with interventions	%
ASO certification	17	60.7
HIV/AIDS training for managers	8	28.6
HIV/AIDS training for peer leaders	12	42.9
HIV/AIDS training for workers	13	46.4
Free condom services available in the establishment	14	50.0
Condom vending machine available in the establishment	11	39.3
Whether or not mobile exhibitions by TBCA/Partner come to this establishment	10	35.7

These seven workplace program variables are summarized into two program inputs by conducting factor analysis (Extraction Method: Principal Component Analysis. Rotation Method: Varimax with Kaiser Normalization. Rotation converged in three iterations). The results (see Table 2) yield the key factors as the ‘management and services’ component (meeting ASO criteria, free condom distribution, condom machine installation and mobile exhibition), and the ‘HIV/AIDS training’ component (training of managers, peer leaders and workers). A multi-level analysis was then conducted to examine the effects of these two program components on HIV/AIDS knowledge, perceived condom accessibility and condom use with regular partners, controlling for individual characteristics including age, sex, marital status and education.

**Table 2.** Rotated component matrix of the factor analysis of the seven HIV/AIDS prevention program interventions requesting for two components.

Interventions	Component 1: Management and services	Component 2: HIV/AIDS training
ASO certification	.935	(.263)
Condom vending machine available in the establishment	.896	(.419)
Free condom services available in the establishment	.883	(.437)
Whether or not mobile exhibitions by TBCA/Partner come to this establishment	.854	(.474)
HIV/AIDS training for managers	(.378)	.922
HIV/AIDS training for peer leaders	(.381)	.921
HIV/AIDS training for workers	(.386)	.919

Note: Coefficients of less than .500 are shown in parenthesis.

Because the data were collected in several factories, it is possible that the error terms on individual cases were correlated within factories. We estimated models using a random effects model to adjust for this correlation. The coefficients of the basic and the random effects models were not significantly different. In this paper, we present the basic models.

## Results

Table 3 shows the demographic and social characteristics, AIDS knowledge, perceived accessibility to condoms and condom use with regular partners at last sex among the study participants. The average age of respondents was 21.7 years (SD = 1.828). 41.2% of the respondents were male, and 55.2% were married. Education among these factory workers varied from primary/middle/secondary school (71.1%) to diploma level (28.9%). HIV/AIDS knowledge among the workers in this study was found to be generally high, with the mean score of 8 out of a total possible score of 9. Perceived condom accessibility or knowledge of where condoms are provided is 94.8%. The level of condom use of these workers with their regular partner at last sex was 26.9% (17.3% for women and 40.7% for men). Among male and female respondents, there was a statistically significant difference between women and men in terms of marital status. Females were currently married than males. On the other hand, males had slightly higher AIDS knowledge score than females, and males are more likely to use condoms with a regular partner than their female counterparts.

**Table 3.** Demographic and social characteristics, AIDS knowledge, perceived accessibility to condoms and condom use among factories workers aged 18–24 who had sex with regular partners, in 28 selected factories undergoing an HIV/AIDS workplace program, Thailand 2008.

Demographic and AIDS related measures	Total	Male	Female
*Mean age* (*N* = 699)	21.7	21.8	21.6
*(SD)*	*(1.828)*	*(1.874)*	*(1.801)*
*Gender*			
For all males and females (*N* = 699)	100.0	36.2	63.8
For those who had sex with regular partners (*N* = 520)	100.0	41.9	58.1
Marital status*** (*N* = 699)			
Married	41.1	26.5	49.3
Not married	58.9	73.5	50.7
*Education* (*N* = 699)			
Primary/Middle/Secondary	71.0	66.4	73.5
Diploma	29.0	33.6	26.5
AIDS knowledge score*** (*N* = 699)			
Score 3 out of 9	0.1	0.0	0.2
Score 5 out of 9	1.3	1.6	1.1
Score 6 out of 9	6.6	5.9	7.0
Score 7 out of 9	21.3	23.3	20.2
Score 8 out of 9	35.8	28.1	40.1
Score 9 out of 9	34.9	41.1	31.4
*(Mean score)*	*(8.0)*	*(8.0)*	*(7.9)*
*Perceived accessibility of condom* (*N* = 699)			
Know where to obtain a condom	93.7	97.2	91.7
Condom use with regular partner***			
(*N* = 520)	26.9	40.7	17.3

***Significant level =< .000.

Tables 4–6 present analysis of the effects of the two components of the program inputs generated from the factor analysis of the seven program input variables. These include (1) The ‘management and services’ component (meeting the ASO criteria, having free condom distribution, having condom vending machines available in the work place, and having mobile HIV/AIDS exhibitions in the workplace), and (2) Implementing the three-level ‘HIV/AIDS training’ for the managers, peer leaders and the workers. The dependent variables include AIDS knowledge (Table 4), perceived accessibility to condoms (Table 5) and condom use with regular partners at last sex (Table 6).

**Table 4.** Multiple regression analysis of the effect of HIV/AIDS workplace prevention program components on HIV/AIDS knowledge among 692 young factory workers (aged 18–24), controlling for individual socio-demographic characteristics.

Independent variables	Unstandardized coefficients		Standardized coefficients	*t*	Sig.	95% Confidence interval for *B*	
	*B*	Std. error	Beta			Lower bound	Upper bound
(Constant)	6.042	.443		13.648	.000	5.172	6.911
Age	.083	.021	.153	3.981	.000	.042	.123
Gender							
Male	.091	.079	.044	1.152	.250	−.064	.246
Female (reference)	–	–	–	–	–	–	–
Education							
Higher than high school	.158	.083	.072	1.891	.059	−.006	.321
High school and lower(reference)	–	–	–	–	–	–	–
Marital status							
Married and ever-married	.110	.079	.055	1.391	.165	−.045	.265
Single (reference)	–	–	–	–	–	–	–
HIV/AIDS workplace prevention program:	.104	.037	.105	2.834	.005	.032	.076
Management and services component							
HIV/AIDS workplace prevention program: Training component	.007	.037	.007	.200	.842	−.065	.080
*R*	.219						
*R* square	.048						
Adjusted *R* square	.040						
Std. error of the estimate	.96859

**Table 5.** Logistic regression analysis of the effect of HIV/AIDS prevention program components on perceived accessibility to condoms among 699 young factory workers (aged 18–24), controlling for individual socio-demographic characteristics.

Independent variables	*B*	S.E.	Wald	df	Sig.	Exp(*B*)	95.0% C.I. for EXP(*B*)	
							Lower	Upper
Age	.029	.089	.102	1	.749	1.029	.864	1.226
Gender								
Male	1.440	.432	11.102	1	.001	4.221	1.809	9.848
Female (reference)	–	–	–	–	–	–	–	–
Education								
Higher than high school	.533	.401	1.770	1	.183	1.705	.777	3.740
High school and lower (reference)	–	–	–	–	–	–	–	–
Marital status								
Married and ever-married	1.090	.377	8.374	1	.004	2.975	1.422	6.226
Single (reference)	–	–	–	–	–	–	–	–
HIV/AIDS workplace prevention program:	1.025	.459	4.987	1	.026	2.788	1.134	6.855
Management and services component								
HIV/AIDS workplace prevention program: Training component	.190	.210	.821	1	.365	1.209	.802	1.824
Constant	1.491	1.912	.608	1	.435	4.443		
−2 Log likelihood	294.161							
Cox and Snell *R* square	.048							
Nagelkerke *R* square	.128

**Table 6.** Logistic regression analysis of the effect of HIV/AIDS prevention program components on condom use with regular partners among 520 young factory workers (aged 18–24), controlling for individual socio-demographic characteristics.

Independent variables	*B*	S.E.	Wald	df	Sig.	Exp(*B*)	95.0% C.I. for EXP(*B*)	
							Lower	Upper
Age	−.079	.063	1.545	1	.214	.924	.816	1.047
Gender								
Male	.602	.235	6.562	1	.010	1.826	1.152	2.895
Female (reference)	–	–	–	–	–	–	–	–
Education								
Higher than high school	−.512	.251	4.154	1	.042	.599	.366	.981
High school and lower (reference)	–	–	–	–	–	–	–	–
Marital status								
Married and ever-married	−.1.922	.260	54.526	1	.000	.146	.088	.244
Single (reference)	–	–	–	–	–	–	–	–
HIV/AIDS workplace prevention program:	.221	.108	4.210	1	.040	1.247	1.010	1.540
Management and services component								
HIV/AIDS workplace prevention program: Training component	.139	.108	1.667	1	.197	1.149	.931	1.419
Constant	1.420	1.364	1.084	1	.298	4.136		
−2 Log likelihood	499.395							
Cox and Snell *R* square	.185							
Nagelkerke *R* square	.269							

The analysis was conducted with the control variables age, gender, education and marital status. Older workers had greater knowledge of HIV/AIDS. The AIDS knowledge score increased by 0.083 for each year of age (*p* < .001) (Table 4). Men tended to have better perceived access to condoms (OR = 4.221 (1.809, 9.848) *p* < .01) (Table 5) and to use condoms with regular partners more than women (OR = 1.826 (1.152, 2.895) *p* < .05) (Table 6). This is probably due to the expectation of a stronger HIV-prevention role for men more than for women. Women’s possession and initiation of use of condom may to be more stigmatized. Table 6 showed that workers with higher than a high school education were less likely to use condoms than other workers (OR = 0.599 (0.366, 0.981) *p* < .05). Table 5 also showed that married workers had higher perceived access to condoms than other workers (OR = 2.975 (1.422, 6.226) *p* < .01). Among those who had regular partners (Table 6), the married workers tended to use condoms less than their unmarried counterparts (OR = 0.146 (0.088, 0.244) *p* < .001).

The analysis also reveals that, after controlling for age, sex, marital status and education, the management and services component of the workplace program was significantly and positively related to HIV/AIDS knowledge (*p* < .01) (Table 4), perceived condom accessibility (OR = 2.788 (1.134, 6.855) *p* < .05) (Table 5), and most importantly, condom use with regular partners (OR = 1.247 (1.010, 1.540) *p* < .05) (Table 6). Although the training component was positively related to the above outcomes the relationships were not statistically significant.

## Discussion

In this study, workers who received the program components had more positive health behaviors. This is consistent with the TBCA program providing cues to action for positive health behaviors. The analysis showed the significance of the management and services component of the intervention program on increased AIDS knowledge, perceived condom accessibility and condom use with regular partners. In the context of the generalized epidemic, the workplace program was proven to be appropriate, practical and successful.

However, despite certain strengths of the study, there are limitations. Because a cross-sectional, rather than a longitudinal survey, was used, the causal relationships found in this study need to be considered with caution. Also, although steps were taken to protect the confidentiality of respondents, the study relies on self-reports of personal behaviors that cannot be verified. In addition, as discussed in the methods section and Table 1, the components of the intervention were not present in many of the factories. For example, the component that was implemented the least (28.6%) was HIV/AIDS training for managers.

Implementing a workplace-based intervention proved to be challenging in a Chinese factory (Qian et al., 2007) although there are successful examples in other locations such as in Botswana (Ronald Hope, 2003). Peer education, a component of many prevention programs was proven to be effective in a workplace program in a country such as Ghana (Wolf, Tawfik, & Bond, 2000). An HIV intervention implemented among factory workers in Ho Chi Minh City also found that AIDS knowledge was improved and condom use increased among factory workers after implementation of the intervention programs (Wolf et al., 2000). The investigators also found that risky sexual behavior of the workers was reduced among those who had participated in a peer-led intervention program (Ngoc et al., 2003). A workplace-based peer education program for industrial workers in Zimbabwe showed a reduced level of HIV infection among workers who participated in the program (Katzenstein et al., 1998). Cost per infection averted compared favorably with other HIV-prevention programs and employers were willing to bear much of the cost of sustaining peer education.

The results in this study do not show the significance of the training component of the workplace program that was one of the foci of this study. Since the trainings of managers, leaders of workers and workers were conducted among a limited number of personnel, the intended diffusion impact on workers did not occur. More personnel may need to be trained to obtain a stronger effect. However, in contrast, the key success of the workplace program in Thailand especially regarding the management and service components found in this study points to the importance at the policy level of acquiring an AIDS standard protocol in the workplace. This policy supports management to provide condom services and AIDS campaigns. This management and service component of the program was found to be crucial for the prevention effort.

A multi-sectoral approach, using unions, management and medical personnel was found to be effective for an HIV-prevention program in a South African sugar mill (Morris, Wilkinson, Stein, & Cheevers, 2001). This multi-sectoral approach in Thailand also produced good results. In Thailand, the engagement of the private sector in HIV prevention was tied to the Ministry of Labor. The endorsement by the Ministry of Labor may have increased the sense of importance of the program for factory owners and managers, facilitating its adoption in the private sector. As shown in Thailand and South Africa, a multi-sectoral approach can facilitate HIV prevention for factory workers.

## Conclusion

Our study showed that prevention program components related to the workplace policy on AIDS, management of available condom services and HIV/AIDS campaigns were significantly related to increased AIDS knowledge, perceived accessibility to condoms and condom use with regular partners among young factory workers. These components of the program may have provided cues to action for the workers. Some revision of the program component related to the training activities and design for scaling up of the workplace intervention should be seriously considered. In particular, policy-makers should investigate the possibility of the engagement of the private sector in HIV prevention. With a multi-sectoral approach and a self-financing strategy, this may enhance their efforts to achieve their zero AIDS infection goals.
